# Cognitive Training in Orthopaedic Surgery

**DOI:** 10.5435/JAAOSGlobal-D-21-00021

**Published:** 2021-03-10

**Authors:** Matthew J. J. Anderson, Alirio J. deMeireles, David P. Trofa, David Kovacevic, Christopher S. Ahmad, Thomas S. Lynch

**Affiliations:** From the Department of Orthopedic Surgery, Columbia University Irving Medical Center, New York, NY.

## Abstract

Over the past two decades, various factors have led to fewer opportunities for hands-on learning in the operating room among orthopaedic surgery trainees. Innovative training platforms using anatomic models, cadaveric specimens, and augmented reality have been devised to address this deficiency in surgical training, but such training tools are often costly with limited accessibility. Cognitive training is a low-cost training technique that improves physical performance by refining the way in which information is mentally processed and has long been used by professional athletes and world-class musicians. More recently, cognitive training tools have been developed for several orthopaedic surgery procedures, but the overall utility of cognitive training in orthopaedic surgery remains unknown.

**Methods:**

The purpose of this study was to review the existing literature regarding the use of cognitive training in orthopaedic surgery and to summarize the results of investigations comparing cognitive training tools with other methods of learning. To that effect, the PubMed and Embase databases were systematically reviewed for articles related to cognitive training in orthopaedic surgery.

**Results:**

Eleven publications met the inclusion criteria, including six randomized controlled trials. Cognitive task analysis and mental rehearsal were the most common forms of cognitive training identified. All 11 publications supported the use of cognitive training in orthopaedic surgery training. In the six randomized controlled trials, the utilization of cognitive training was associated with notably improved surgical performance and increased knowledge compared with traditional methods of learning.

**Discussion:**

Based on the limited evidence presented in this review, cognitive training represents a promising, low-cost adjunct to traditional orthopaedic surgery training. Further efforts should be directed at developing and evaluating additional cognitive training tools for orthopaedic surgery trainees.

The orthopaedic surgery training environment is under increasing pressure because of a combination of factors including resident work-hour restrictions, increasing malpractice claims, efforts to improve patient safety, greater emphasis on efficiency in the operating room (OR), the popularization of minimally invasive surgical approaches, and most recently, the COVID-19 pandemic.^1–3^ For orthopaedic surgery trainees, the net result of these various factors has been decreased hands-on training in the OR.^4,5^ However, residents are still expected to demonstrate proficiency in core procedures as defined by the Accreditation Council for Graduate Medical Education.

In light of the aforementioned changes in the orthopaedic surgery training landscape, the importance of preoperative preparation is greater than ever because trainees must maximize the utility of OR experiences. At the same time, a growing recognition exists of the importance of cognitive ability in the execution of surgical procedures.^6–8^ This has led to a paradigm shift in surgical training, whereby trainees are encouraged to focus less on acquiring motor skills and more on understanding the reasoning and decision-making behind each procedural step.^6–8^ In this process, various cognitive training tools have been devised and adapted from other fields to supplement traditional surgical training.

Cognitive training is a method of improving physical performance by refining the manner in which information is mentally processed and manipulated.^7^ The technique is based on the premise that the motor system is part of a cognitive network—a theory supported by functional neuroimaging studies showing that cognitive training and physical performance share common neuronal pathways.^9,10^ Cognitive training can be used for the acquisition of new motor skills, the maintenance of existing skills, or the transference of existing skills to new tasks^11^ and has long been used with great efficacy among professional athletes, musicians, pilots, and military personnel.^7,12–14^ More recently, cognitive training has demonstrated great promise as a cost-effective, efficacious training adjunct in several surgical specialties including general surgery, obstetrics and gynecology, otolaryngology, and vascular surgery.^12,15,16,17,18^

Although several methods of cognitive training exist, cognitive task analysis (CTA) and mental rehearsal (MR) are perhaps the most studied in the field of surgical training. CTA is a systematic process by which experts break down a complex task into discrete steps. In addition to simply describing the steps, however, emphasis is placed on the decision-making required to successfully progress through the task. Experts are asked to provide the rationale behind each step while identifying common pitfalls and sources of errors. MR, also known as cognitive rehearsal or mental practice, is a training technique that involves deliberate visualization of a task in the absence of overt physical movement. Typically preceded by relaxation exercises, MR uses imagery to visually rehearse a task before its physical performance.

The purpose of this study was to review the existing literature regarding the use of cognitive training in orthopaedic surgery and to summarize the results of investigations comparing cognitive training tools with other methods of learning for orthopaedic procedures.

## Methods

The PubMed and Embase databases were searched for scientific articles related to cognitive training in orthopaedic surgery published between January 2010 and June 2020. The search strategy was designed in conjunction with the authors' institution's clinical informationist and consisted of dividing the search terms into three silos: (1) terms related to cognitive training, (2) terms related to orthopaedic surgery, and (3) terms related to surgical training (Table 1). The Boolean operator “OR” was used to separate words within each category, whereas the Boolean operator “AND” was used to link the three categories.

**Table 1:** Key Terms Used During the Database Search

Cognitive Training Silo	Surgical Field Silo	Surgical Training Silo
Auditory imagery	Orthopaedic^a^	**Education**
**Behavior therapy**^a^	**Orthopaedic nursing**	Education^a^
Brain training	**Orthopaedic procedures**	**Education, medical, undergraduate**
**Cognition**	**Orthopaedic surgeons**	Intern^a^
**Cognitive behavioral therapy**^a^	Orthopaedic^a^	**Internship and residency**
Cognitive imagery	**Orthopaedics**	Learn^a^
Cognitive task analysis		**Learning**
Cognitive training		Medical student^a^
**Imagery, psychotherapy**^a^		Residency^a^
Mental imagery		Resident^a^
Mental practice		Students, medical
Mental preparation		**Teaching**
Mental rehearsal		Teaching
Mental skill		Trainee^a^
Mental therapy		Training
Mental training		
Motor imagery		
Motor practice		
Motor training		
Olfactory imagery		
Visual imagery		
Visual therapy		
Visual training		

MeSH terms are presented in bold.

a Including an asterisk at the end of a search term allows for truncation, a method of broadening a search by using a symbol to replace any combination of letters or words.

### Selection Criteria

Only studies that met the following inclusion criteria were considered: (1) studies had to evaluate or describe the use of CTA and/or MR as a method of surgical training, (2) study participants had to include orthopaedic surgeons or the procedure/skill under investigation had to be relevant to orthopaedic surgery, (3) studies had to be written or translated into English, and (4) full-text articles had to be available for review. Studies assessing other surgical training platforms (e.g., cadaver models, animal models, bench models, and virtual reality) with an explicit cognitive training component were considered for inclusion.

### Article Screening and Selection

The database search was done by two orthopaedic surgery residents (M.J.J.A. and A.J.d.M.) with the assistance of a clinical informationist. Publications were initially screened by title and then by abstract. The full-text descriptions of the remaining articles were then assessed to determine whether all inclusion criteria were met. The reference sections of included articles were manually reviewed to identify additional studies not captured during the database search. Data extraction was done simultaneously by two authors (M.J.J.A. and A.J.d.M.) to maximize accuracy. Any discrepancies during the database search or data extraction processes were resolved via discussion between the two authors.

### Data Analysis

Basic study characteristics including author(s), year of publication, study design, level of evidence, number of participants, and level of training of participants were abstracted for all studies. Each randomized controlled trial (RCT) was graded according to the Jadad scale, which ranges from zero (poorly designed trial) to five (rigorous study methodology).^19^ The Jadad score is calculated based on study randomization, blinding, and documentation of participant withdrawal.^19^ Details pertaining to the cognitive training tool under investigation, the task participants were asked to do, the control group, methods of assessment, and outcome measures were collected for each study. Because outcome measures varied between studies, a meta-analysis could not be done. Microsoft Excel (Microsoft) was used to aggregate all raw data and do basic statistical analyses.

## Results

### Literature Search Results

**Figure 1:**
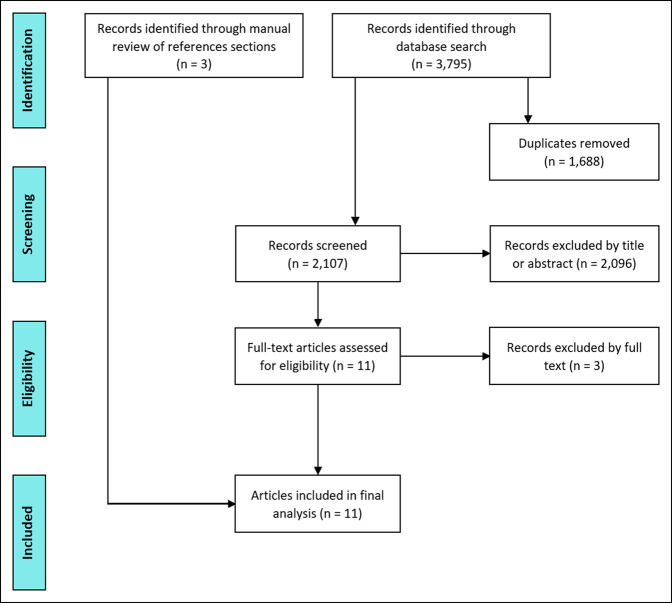
Flow diagram outlining the systematic review process.

The literature search produced 3,795 results, of which 3,787 (99.8%) were excluded as outlined in Figure 1. The reference sections of the remaining eight studies were manually reviewed, resulting in three additional studies that met all inclusion criteria, for a total of 11 publications. Nine studies (82%) focused on CTA exclusively, one study (9%) discussed both CTA and MR, and one study (9%) addressed the use of MR alone. Six publications were RCTs comparing CTA with other methods of surgical training, one was a prospective cohort study comparing the performance of expert and novice surgeons using a CTA tool, one was a survey-based study that asked participants to evaluate the usefulness of a CTA tool, one was a descriptive study outlining the process of developing a CTA tool, one was an interview-based study of expert orthopaedic surgeons regarding the use of MR in preoperative planning, and one was an editorial piece describing the utility of cognitive training in preventing surgical skill decay. The individual article characteristics and outcomes are summarized in Table 2.

**Table 2:** Overview of Studies Investigating the Use of Cognitive Training in Orthopaedic Surgery

Authors	Year	Cognitive Training	Design Overview	Participants	Overall Findings
Amer et al^20^	2017	CTA	Prospective RCT of CTA tool vs video lecture for carpal tunnel release	100	CTA cohort demonstrated notably greater procedural knowledge
Bhattacharyya et al^21^	2017	CTA	Prospective RCT of CTA tool vs no additional learning materials for knee arthroscopy	16	CTA cohort did notably better during knee arthroscopy simulation
Bhattacharyya et al^22^	2018	CTA	Prospective RCT of CTA tool vs surgical technique manual for antegrade femoral IMN	22	CTA cohort demonstrated notably greater procedural knowledge
Bunogerane et al^23^	2018	CTA	Prospective RCT of CTA tool vs textbook chapter for tendon repair	27	CTA cohort demonstrated notably better technical skills during tendon repair simulation and greater procedural knowledge
Ibrahim et al^24^	2015	MR	Interview-based study of expert surgeons regarding the use of MR in preoperative planning	9	Expert orthopaedic surgeons frequently use MR to prepare for surgical procedures
Kelc et al^25^	2020	CTA, MR	Perspective piece on the benefits of cognitive training for orthopaedic surgeons	NA	Cognitive training can be used to prevent skill delay in orthopaedic surgeons who are temporarily unable to operate
Levin et al^26^	2018	CTA	Prospective survey-based study on the perceived utility of CTA tools	14	CTA tools thought to improve understanding and accelerate learning of basic orthopaedic procedures among trainees
Logishetty et al^27^	2020	CTA	Prospective RCT of CTA tool vs operation manual and video for anterior approach THA	36	CTA cohort did notably better on a simulated anterior approach THA and demonstrated greater procedural knowledge
Sugand et al^28^	2015	CTA	Prospective validation study of CTA tool comparing expert and novice surgeons for femoral IMN	49	Experts notably outperformed novices to demonstrate construct validity
Vestermark et al^29^	2019	CTA	Prospective RCT comparing CTA tool vs surgical reference guide for robotic-assisted UKA	12	CTA cohort demonstrated notably greater procedural knowledge and a trend toward better retention at three weeks
Yeung et al^30^	2017	CTA	Instructional report describing the process of creating CTA tools for basic hand procedures	28	CTA can be used to generate online tools that enhance the acquisition of basic hand procedures

CTA = cognitive task analysis, IMN = intramedullary nailing, MR = mental rehearsal, NA = not applicable, RCT = randomized controlled trial, THA = total hip arthroplasty, UKA = unicompartmental knee arthroplasty

### Randomized Controlled Trials Assessing Cognitive Task Analysis

The six RCTs had an average Jadad score of four and included a total of 213 participants, of whom 50.2% (n = 107) underwent cognitive training and 49.8% (n = 106) served as controls (Table 3). The participants included 4 fellows (1.9%), 87 residents (40.8%), and 122 medical students (57.3%). In three studies, the CTA tool under investigation was a Touch Surgery module(s). Touch Surgery is an interactive mobile-based application that combines CTA with virtual reality, allowing users to rehearse the steps of various surgical procedures. In the remaining three studies, novel web-based CTA tools were developed that involved a combination of written information, video clips, and audio recordings.

**Table 3:** Study Characteristics of Prospective Randomized Controlled Trials Comparing the Use of CTA Tools With Other Methods of learning

Authors	Year	Jadad Score	Participants (n)	Procedure	Cohorts	Assessment Method(s)	Outcome Details
Amer et al^20^	2017	3	Medical students (100)	Carpal tunnel release	Touch Surgery module vs video lecture	21 multiple-choice questions	CTA cohort had a significantly higher mean test score (89.3% vs 75.6%, *P* < 0.05); usefulness of CTA tool rated as very high (mean 4.7 of 5)
Bhattacharyya et al^21^	2017	5	Residents (16)	Diagnostic knee arthroscopy	IKACTA tool vs no additional learning materials	Simulated knee arthroscopy graded using the ASSET global rating scale	CTA cohort did significantly better on a high-fidelity, phantom-knee simulator as measured on the ASSET scale (mean 19.5 vs 10.6 points, *P* = 0.002); all participants rated the CTA tool as useful
Bhattacharyya et al^22^	2018	5	Medical students (22)	Antegrade femoral IMN	IFINCTA tool vs surgical technique manual	Touch surgery assessment tool for (1) patient positioning and preparation, (2) femoral canal preparation, (3) proximal locking, and (4) distal locking and closure	CTA cohort scored significantly higher on all four touch surgery modules (mean 80 vs 60 points for patient positioning and preparation, 79 vs 58 points for femoral canal preparation, 77 vs 67 points for proximal locking, and 82 vs 63 points for distal locking and closure, *P* ≤ 0.001 for all); all participants agreed that the CTA tool was beneficial
Bunogerane et al^23^	2018	4	Residents (27)	Tendon repair	Touch Surgery module vs textbook chapter	Simulated tendon repair using a real tendon, multiple-choice questions	CTA cohort did better on tendon repair simulation (mean 89.7% vs 63.4%, *P* < 0.001) and multiple-choice questions (mean improvement from baseline of 38.6%, *P* < 0.001 vs 15.9%, *P* = 0.304); 92.3% of participants rated the CTA tool as useful
Logishetty et al^27^	2020	5	Residents (36)	Anterior approach THA	Imperial College Digital Learning Hub web-based tool vs operation manual and video	Simulated anterior approach THA using augmented reality, 10 multiple-choice questions	CTA cohort did simulated anterior approach THA faster (mean 28 vs 38 minutes, *P* < 0.005) with fewer errors (mean 29 vs 49 instances) and required fewer prompts (13 vs 25 instances); acetabular cup orientation was more accurate in the CTA cohort (mean combined error of 16 vs 24°, *P* < 0.005); CTA cohort did better on multiple-choice questions (mean 6 vs 4 points, *P* < 0.005); 97.2% of participants rated the CTA tool as useful
Vestermark et al^29^	2019	2	Fellows (4), residents (8)	Robotic-assisted UKA	Touch Surgery modules vs surgical reference guide	25 multiple-choice questions	CTA cohort demonstrated a significantly greater mean improvement in test score relative to baseline (22%, *P* = 0.001 vs 10%, *P* = 0.13) and a trend toward better recall at three weeks (*P* = 0.09)

ASSET= Arthroscopic Surgical Skill Evaluation Tool, CTA = cognitive task analysis, IFINCTA = Imperial Femoral Intramedullary Nailing Cognitive Task Analysis, IKACTA = Imperial Knee Arthroscopy Cognitive Task Analysis, IMN = intramedullary nailing, THA = total hip arthroplasty, UKA = unicompartmental knee arthroplasty

The surgical procedure done by the participants varied by study and included anterior approach total hip arthroplasty, diagnostic knee arthroscopy, antegrade femoral intramedullary nailing, carpal tunnel release, robotic-assisted unicompartmental knee arthroplasty, and tendon repair. The learning materials provided to the control group also varied by study and included a surgical technique guide (2), a surgical technique guide plus a video (1), a slide show with audio (1), a textbook (1), and no additional learning materials in one study. Assessment methods included graded simulation using a model (1), graded simulation using a real specimen plus multiple-choice questions (1), graded simulation using virtual reality plus multiple-choice questions (1), multiple-choice questions alone (2), and a Touch Surgery assessment tool (1).

In outcomes, the overall effect of CTA was noted to be positive in all six studies. More specifically, utilization of CTA was associated with improved performance and shorter surgical time during simulated procedures^20–22^ and increased knowledge as measured by multiple-choice questions.^21–25^ Vestermark et al^25^ noted a trend toward better retention of procedural knowledge in the CTA group compared with the control group at three weeks, but the difference was not statistically significant (*P* = 0.09). In addition, five of the six studies asked participants to evaluate the utility of the CTA tool under investigation, and all five CTA tools were graded as useful.

### Additional Cognitive Task Analysis Studies

Levin et al^26^ assessed the utility of Touch Surgery modules among 14 first-year orthopaedic surgery residents and found that 71.4% of participants felt that the application improved their baseline understanding of the procedures (open reduction and internal fixation of an ankle fracture and lag screw fixation). To assess the construct validity of Touch Surgery modules for intramedullary femoral nailing, Sugand et al^27^ compared the performance of medical students (novices) with that of orthopaedic surgery fellows and attendings (experts). The experts did significantly better than novices on all four modules under investigation (*P* < 0.001), suggesting construct validity. In addition, both cohorts agreed that the modules were useful for preoperative rehearsal. The final CTA study by Yeung et al^30^ described the process of developing CTA-based multimedia videos for basic surgical skills.

### Mental Rehearsal Studies

The only study to assess the use of MR in orthopaedic surgery was an interview-based study of the nine senior orthopaedic traumatologists.^29^ All surgeons reported extensive use of mental imagery in the context of preoperative preparation.^29^ MR consisted of pure visualization for some surgeons, whereas others used a combination of visual and tactile sensory modalities.^29^ The article by Kelc et al^30^ advocates for the use of MR and CTA to prevent skill decay among orthopaedic surgeons who are temporarily unable to operate.

## Discussion

This is the first systematic review to examine the use of cognitive training in orthopaedic surgery. Based on the limited number of studies identified, the incorporation of cognitive training tools into orthopaedic surgery training curriculum remains in its infancy. However, the few studies that have critically assessed the utility of cognitive training in orthopaedic surgery, specifically CTA, demonstrated notable benefit across all levels of training relative to traditional methods of learning (eg, textbooks, surgical technique guides, slide shows, and videos). Considering the proven efficacy of cognitive training in other fields, the mounting limitations on hands-on surgical training opportunities, and the relatively inexpensive, accessible, and safe nature of most cognitive training tools developed thus far, additional research is warranted to further elucidate ways in which cognitive training can be better integrated into orthopaedic surgery training.

Traditionally, surgical training has largely focused on the development of specific motor skills and the memorization of procedural steps through observation and repetition.^12,31^ However, the intraoperative decision-making that enables expert surgeons to successfully and safely complete procedures is often more difficult to teach and arguably more important in the development of surgical trainees.^12,31^ Moreover, a cognitive component of motor skill acquisition exists that is well supported by neuroimaging studies, further highlighting the importance of cognitive development among novice surgeons.^7,9,12,32^ Spencer^32^ went so far as to propose that cognitive ability underlies 75% of surgical training, whereas mechanical ability comprises only 25%. Fortunately, recent advances in technology have facilitated the development of various training platforms that emphasize the development of cognitive skills. This review supports the notion that such novel platforms based on cognitive training principles, specifically MR and CTA, can lead to improved surgical performance while minimizing risk to patients.

CTA was first described in the 1980s and has been used with great success as a cost-effective training technique among pilots, military personnel, musicians, and Olympic athletes.^12,27,33^ More recently, CTA tools have been developed and studied for various surgical procedures, ranging from laparoscopic appendectomy to open cricothyrotomy.^34,35^ Wingfield et al^12^ conducted a systematic review assessing the use of CTA as a training tool across all surgical specialties and found that CTA improved surgical outcome parameters in 12 of 13 studies (92.3%). The authors concluded that CTA can notably improve surgical performance and efficiency among surgeons at all levels of training.^12^ The results of the current systematic review are equally supportive of the use of CTA as a training methodology in orthopaedic surgery, with all six studies (100%) comparing CTA with other methods of learning demonstrating notably better performance in the CTA cohort.

It has been estimated that expert surgeons omit approximately 70% of relevant knowledge when teaching a surgical procedure, which has been attributed to a process of automation, whereby certain aspects of a procedure become second nature and no longer require conscious thought.^36^ CTA tools are often developed using a modified Delphi technique, in which input from several experts is combined to generate a comprehensive list of technical steps, cognitive decision points, and common errors.^21,22,27^ In this manner, CTA tools enhance learning by countering the phenomenon of knowledge automation in addition to providing rationale behind critical steps and offering solutions to common obstacles.^12^ Moreover, CTA tools often combine written text with audiovisual content in an online or digital platform to further facilitate learning and offer increased accessibility. Because of these various attributes, CTA offers distinct advantages over most traditional and novel learning modalities and warrants serious consideration as an integral component of the orthopaedic surgery training curriculum moving forward.

Similar to CTA, MR has long been used by high-level athletes and world-class musicians.^13,14^ More recently, the utility of MR as a training technique has been established in numerous surgical specialties including general surgery, vascular surgery, obstetrics and gynecology, and otolaryngology.^15,16,17,18,37^ For instance, Arora et al^18^ observed novice surgeons doing simulated laparoscopic cholecystectomies using a virtual reality platform and found that participants who did 30 minutes of MR before each simulated procedure had superior technical performance and improved mental imagery compared with participants who viewed an online lecture. The incorporation of MR into orthopaedic surgery training, on the other hand, has lagged behind other surgical specialties. As revealed by the current systematic review, only two studies have addressed the role of MR in orthopaedic surgery over the past 10 years. Moreover, neither of these two studies formally evaluated the potential benefits of MR in any meaningful way.

Although MR has not been well studied as a formal component of orthopaedic surgery training, many experienced orthopaedic surgeons routinely use MR during preoperative preparation.^29^ Interestingly, however, previous research suggests that MR may be most beneficial if used early during surgical training.^11,15^ In addition to assisting with the acquisition of basic motor skills and learning the steps of various procedures, MR has several potential psychological benefits that may prove especially helpful for young surgical trainees. For instance, MR can notably increase self-confidence and reduce subjective stress.^37,38^ Doing MR before simulated surgery has even been shown to lower objective measures of stress including heart rate and salivary cortisol.^37,38^ Although the potential benefits of incorporating MR into surgical training have largely been documented in specialties other than orthopaedic surgery, no reason exists to think that MR cannot be similarly beneficial in orthopaedic surgery training. In fact, given the standardized nature of many orthopaedic procedures, the utility of MR may be even greater in orthopaedic surgery compared with other specialties.

This review has several limitations that warrant further discussion. Overall, the small number of studies addressing cognitive training in orthopaedic surgery limits our conclusions and illustrates the need for additional research in this area. In addition, the cognitive training tools assessed, the tasks/procedures done, and the outcome measures varied notably by study, which obfuscated aggregate analysis. Although the observed variability between studies may suggest that various cognitive training tools can be used effectively for a range of orthopaedic procedures with diverse potential benefits, it is possible that certain procedures are more amenable to cognitive training techniques. Furthermore, the benefits of cognitive training may vary based on other variables, such as training level. Future studies should aim to better define the optimal role of cognitive training within orthopaedic surgery training. It should also be noted that numerous forms of cognitive training exist beyond those considered in this review. In addition, other surgical training platforms outside the realm of cognitive training, such as virtual reality, have shown notable promise. However, many such platforms are costly and require special equipment, potentially limiting access and restricting widespread adoption.^39,40^ Nonetheless, integrating cognitive training principles into platforms with a hands-on component may represent the future of surgical training.

## Summary

Current orthopaedic surgery trainees have fewer opportunities for hands-on learning in the OR because of various factors including work-hour restrictions, medicolegal issues, and the COVID-19 pandemic. Predicated on demonstrated success among elite athletes, professional musicians, and surgical trainees in other specialties, innovative training tools rooted in cognitive training principles have been devised to address this deficiency in orthopaedic surgery training. As illustrated in this review, an overall paucity of literature assessing the utility of integrating such tools into the orthopaedic training curriculum exists. However, all the studies identified in this review were found to support the use of cognitive training among orthopaedic surgery trainees, suggesting that cognitive training may lead to better surgical performance and increased knowledge compared with other traditional methods of learning. Further efforts should be directed at developing and evaluating additional cognitive training tools for orthopaedic surgery trainees.
